# Narrowing the Gap in Managing the Lymphedema of Lymphatic Filariasis

**DOI:** 10.4269/ajtmh.24-0565

**Published:** 2024-09-17

**Authors:** James W. Kazura

**Affiliations:** Case Western Reserve University, Cleveland, Ohio

Since its inception in 2000, the Global Program to Eliminate Lymphatic Filariasis has made remarkable progress in reducing and stopping the transmission of lymphatic filariasis (LF) through programs of mass drug administration.^1^ However, an important clinical and public health gap in LF care remains among patients with residual pathology due to prior infection. These patients include an estimated 15 million people with lymphedema of the extremities.^2^ This Supplemental issue includes six papers that describe the outcomes of clinical trials that address this gap in knowledge.

Current management of LF lymphedema involves careful maintenance of local hygiene of the extremities and prevention and treatment of coexisting bacterial or fungal infections of the skin. The WHO Essential Package of Care (EPC) reflects this approach, and does appear to be helpful.^3^ Nevertheless, a broader evidence base and additional therapeutic and preventative tools are required to improve lymphedema management and support its continued use.

Previous studies in experimental animal models of LF, and in LF patients with lymphedema, have suggested that treatment with doxycycline might be beneficial. After first reviewing the pathogenesis and current management approaches to lymphedema,^4^ this Supplement describes the results of an experimental protocol carried out in five countries to evaluate the potential benefit of a course of doxycycline to improve the impact of the WHO EPC on LF lymphedema management. Coordinated placebo-controlled, double-masked, randomized clinical trials^5^ were conducted in three African countries (Mali,^6^ Ghana,^7^ and Tanzania^8^) and two countries in South Asia (Sri Lanka,^9^ and India^10^). Each site included between 200 and 400 patients with stage 1–3 lymphedema, and was randomized to receive either the standard-of-care based on the EPC or the EPC plus a 6-week course of daily doxycycline. The goal was to test whether doxycycline could be a therapeutic adjunct to EPC to prevent worsening and promote improvement in lymphedema.

In all, more than 1,400 patients with LF lymphedema were monitored intensively for 2 years,^5^ recording clinical observations that included signs and symptoms of lymphedema and adenolymphangitis, physical and volumetric measurements of lymphedema, qualitative assessment of well-being, compliance with provided medications, and adherence to EPC recommendations. The primary endpoint at 2 years was scored as “improved,” “not improved”, or “worsened” lymphedema. Overall, 16–40% of patients showed “improved” lymphedema, whereas only a small number showed “worsened” lymphedema. A decreased frequency and duration of adenolymphangitis events and improved, qualitatively assessed well-being were also observed in study participants from all of the sites. Notably, however, these positive changes were nearly identical in patients randomized to treatment with doxycycline versus those receiving the placebo. Thus, the results of these studies, while appreciably strengthening the evidence base for the effectiveness of EPC in managing LF lymphedema, did not identify additional clinical benefit from adjunctive treatment with doxycycline. Modest variability in outcomes from the different study sites and the reasons for these differences are being analyzed to gain deeper insight into the management of LF lymphedema and to enhance uptake of best practices by patients and the local health systems that serve them.

There are notable features of these multi-country studies that merit additional comment with respect to their health significance. Unlike previous Morbidity Management and Disability Prevention studies of LF that have frequently been considered anecdotal and based on “soft science,” the current studies have a high degree of scientific rigor and detailed study designs, with identical clinical trials conducted in five countries over 2 years. The results show clearly that the WHO EPC package is an effective strategy to decrease the severity of lymphedema in many LF patients. The similarity of results across five countries, where there are inherent differences in healthcare systems, socioeconomic environments, ethnicity, and risks of secondary infections, lends credence to the studies’ basic conclusions and are a credit to the expert in-country teams that conducted this research. Also important among the lessons learned from the study are the need to develop more sensitive methods to monitor and quantify lymphedema than the simple three-stage system currently in use, and to create a more holistic approach to health and disability management, as exemplified by WHO Disability Assessment Schedule 2.0. Further, as noted in the first paper of this Supplement,^4^ despite confirmation of the value of the WHO EPC, continued needs include improvement in lymphedema care at the individual level and development of a workable strategy for sustainable program maintenance by patients and local public health systems.
